# Concerns and reflections on the second wave of COVID-19 in Argentina: A perspective by general surgery residents

**DOI:** 10.1016/j.amsu.2021.102860

**Published:** 2021-09-14

**Authors:** René M. Palacios Huatuco, Julian E. Liaño, Gonzalo M. Bono, Gabriel A. González Vargas, Lucas Panichelli, Rafael Pereyra Ferrero, María S. Ponce Beti

**Affiliations:** Department of General Surgery, Clínica Universitaria Reina Fabiola, Universidad Católica de Córdoba, Oncativo 1248, Córdoba Capital, Argentina; Department of General Surgery, Clínica Universitaria Reina Fabiola, Universidad Católica de Córdoba, Oncativo 1248, Córdoba Capital, Argentina; Department of General Surgery, Instituto Médico Río Cuarto, Hipólito Yrigoyen 1020, Río Cuarto, Córdoba, Argentina; Department of General Surgery, Policlínico Privado San Lucas S.A., Mitre 930. Río Cuarto, Córdoba, Argentina; Department of General Surgery, Hospital Privado Universitario de Córdoba, Av. Naciones Unidas 346, Córdoba Capital, Argentina; Department of General Surgery, Hospital Privado Universitario de Córdoba, Av. Naciones Unidas 346, Córdoba Capital, Argentina; Department of General Surgery, Hospital Militar Regional Córdoba, Av. Cruz Roja Argentina 1174, Córdoba Capital, Argentina

**Keywords:** Coronavirus, Crisis, South America, Resident education, Resident safety

## Abstract

In Argentina, the second wave of COVID-19, which started in May, clearly differentiates us from the rest of the Latin American countries, whose current growth may be the announcement of the expected autumn-winter expansion. There is a lot of uncertainty about how the pandemic will evolve, which contrasts with the expectations that had been generated in society after the end of the confinement, both of the control of the health system and access to effective vaccines. Thus, a group of surgeons in training raised a series of concerns concerning the critical situation that we are facing.

## 1 Introduction

The second wave of the pandemic in Argentina confirms the lack of control over it, it is evidence that impacts society, the economy, and health. Health professionals must be at the service of overcoming this deep and complex crisis that we face. To do this, a series of barriers must be overcome: lack of knowledge, imperfect institutional frameworks, a tense political climate, and an unstable social conscience.

In this scenario, the Association of Residents and Concurrent Surgery of Cordoba expresses its concern about the reactivation of COVID in Argentina in recent weeks. Thus, we ask ourselves some questions regarding the arrival of this new wave and its impact on the health system, mainly in surgical practice, which is our responsibility as surgeons in training.

### 1.1 How to define the current situation from the point of view of pandemic control?

In Argentina, the second wave of COVID-19 began in May 2021, registering a 50% increase in cases compared to previous weeks, reaching a maximum of almost 40.000 daily cases, being considered the second country in Latin America with the highest number of COVID-19 cases by country [1]. The number and extent of outbreaks mean a clear risk of overflowing of the local control capacity.

In this scenario, the country returned to strict confinement due to the worsening of the epidemiological situation. With the restrictions, the government was trying to gain time while vaccination progressed, which until now is progressing more slowly than it was initially anticipated, with less than 20% of the population vaccinated with the first dose, out of a population of 45 million. However, these restrictions have divided the Argentine population, but among medical personnel, there is a consensus: “there is a real risk of sanitary collapse, and the circulation of people must be further restricted to avoid it".

The vaccination campaign tries to reduce the cases, but even so, those who are on the front line of the battle against the coronavirus despair at the lack of compliance with the measures decreed by the government. Moreover, there is poor preparation of the health system which falls into a situation even more critical than the first wave.

### 1.2 How was the impact of the second wave perceived on surgical activity?

We are faced with a great trend of patients who come to the doctor's appointment with diseases in advanced stages and with a greater need for surgical treatment. Long waiting time for surgery due to confinement leads to worse postoperative results.

Most of the healthcare centers restarted the protocols that were applied in the initial stage of the pandemic. Thus, the Argentine Association of Surgery [2], in the attempt to face this adversity, recommended the performance of emergency and oncologic surgeries and/or all those that, at the surgeon's discretion, are unpostponable by putting the patient's evolution at risk.

The increased occupancy of beds for COVID-19 patients is worrying, affecting a decrease in the number of more complex surgical procedures. On the other hand, only hospitals with more resources were able to manage another position, beginning to perform elective surgeries with the pertinent care and hygiene measures. Understanding the concern of residents of surgical programs, we conducted a cross-sectional study during the first wave in which the exposure of the number of residents in the operating room was limited and the number of surgical interventions performed showed a significant decrease, which generated a reduction in the surgical experience, mainly in junior residents (PGY - 1 and PGY - 2) [3].

In this scenario, with the decrease in surgical cases, the education and surgical training of surgeons in training has suffered. An event that is repeated and through which we had already passed during the first wave [3]. Therefore, it is necessary to develop surgical skills in controlled simulation environments, which emerges as a complement at the same time that surgeons are dedicated to the care of patients. The simulation allows the training of the different skills that make up the spectrum of professional competence. Achieving technical, cognitive and behavioral skills are goals that simulation-based training can achieve [4].

### 1.3 How to decide the ideal time for surgery?

Several surgical societies worldwide issued recommendations and clinical guidelines to generalize safe surgical practice during the onset of the pandemic. However, we are in a period of adaptation, in which the priority for the reintroduction of elective surgeries to be achieved must be the safety of the patient and the professionals involved.

In patients with suspected COVID-19, it was proposed to wait for the result of a PCR test, but in certain situations, there is no immediate availability or time to wait for the result, in such cases a pre-operative CT scan could be considered. However, the use of CT as a method of screening or diagnosis of COVID-19 is not recommended by the American College of Radiologists [5], who suggest using it in symptomatic, hospitalized patients and with indications for it. The use of serological tests was also suggested, although a negative result during the first seven days of illness cannot rule it out.

In Argentina, the occupation of beds in intensive care by patients with coronavirus was higher compared to the first wave, so some institutions opted to operate only low-risk patients and surgeries of medium and low complexity, with the aim that hospital discharge was the same day of the procedure. Thus, the “ideal” time for elective surgeries remains controversial and depends on each case and the hospital center.

### 1.4 What provisions supported hospital safety?

This pandemic forced us to reorganize and reallocate resources, which led to a reduction and prioritization of surgical activities around the world.

During the first wave, there was a trend towards a more conservative attitude, due to the lack of availability of ultrafiltration systems, the shortage of personal protective equipment (PPE), the absence of vaccines, and the impossibility of performing routine tests on all patients. The response of the health system remains reactive, rather than proactive; although a better preparation of the centers has been achieved, allowing health personnel to feel safe and surgical care of COVID-19 patients can be increased.

There is still controversy regarding the surgical approach, and it has not been elucidated if the use of laparoscopy produces a greater risk of transmission of COVID-19, but it is known that when the air contained in the pneumoperitoneum is released, it could be contaminated and increase the risk of contagion, however, there is no evidence to prefer open surgery [6].

### 1.5 How could the situation of non-COVID patients be described for those who have pending medical examinations, procedures, or interventions and with accumulated delays?

The important adaptation of the entire health system is worrying, as well as the suspension of scheduled surgeries, the transformation of resuscitation posts for critical care, the habilitation of hospital areas to expand hospitalization or intermediate care, and the reassignment of professionals to care for patients outside their usual scope of practice.

The postponement of procedures in non-COVID patients and the accumulation of patients on waiting lists burden hospitals and health professionals [7]. Having to combine COVID and non-COVID patients with parallel circuits means a reduction in the real healthcare capacity.

Elective surgery will be the visible tip of the iceberg, but even in this one, a part will not appear because the indication itself will be diminished due to delays in diagnosis or adjustments in the indication criteria.

So that non-COVID patients do not continue to be deprioritized, reinforcement measures need to be included in the strategies and contingency plans to assist in the delay that is accumulated in new patients, in the control and review of chronic patients or their monitoring.

For all these reasons, we must make the population and social agents aware that COVID strategies must include elements of reactivation of care for all patients and pathologies, particularly those that accumulate risks and severity.

### 1.6 Did the awareness of the population, the social and political climate evolve concerning the first wave of the pandemic?

Compared to the first wave, it seems that social consciousness has declined, it went from applause in total confinement to a growing indignation of the population at the inability to return to daily life. Adherence to the recommendations to avoid the transmission of COVID-19 is more complex and difficult with the “new normality”. Moreover, that social commitment has weakened over time, with a relaxation of physical distance and protection measures. In addition, much of the “personal” fear has been lost, with the belief that the virus is less contagious and deadly [8].

With the advent of COVID-19 vaccines, something may be starting to change during the second wave. However, this progress was overshadowed when the “VIP Vaccination” scandal was uncovered on February 19, in which citizens were vaccinated at the Ministry of Health of Argentina; but due to the limitations established in the vaccination protocol, they should have not received such vaccinations yet. A fact that has arisen in other Latin American countries.

There is a clear feeling of disappointment among health personnel, which overlaps the fatigue of several months of intense work and exhausting days. This disappointment is also directed to the behavior of political and institutional leaders.

Healthcare reinforcement has been uneven and precarious, and there is still a lack of common leadership in everyone's health response to COVID-19.

Many actions could be launched in the short term, but others require reforms and time. Health authorities must work collaboratively to create a commonly accepted and respected framework for the standardization of public health and surveillance actions against COVID-19 and thus increase the reliability of our actions before the population.

## Ethical approval

Ethical approval not required.

## Sources of funding

No source to be stated.

## Author contribution

Study conception and design: RPH, MPB. Acquisition of data: All authors. Analysis and Interpretation of data: All authors. Drafting of Manuscript: All authors. Critical Review and approval of the final version: All authors.

## Registration of research studies

1. Name of the registry: N/A *.

2. Unique Identifying number or registration ID: N/A *.

3. Hyperlink to your specific registration (must be publicly accessible and will be checked): N/A *.

* Not applicable.

## Guarantor

Gabriel A. González Vargas: gagonzalezvargas@gmail.com.

## Consent

Not applicable.

## Declaration of competing interest

There is no conflict to be declared.
